# Editorial: Sirtuinome Rewiring to Hijack Cancer Cell Behavior and Hamper Resistance to Anticancer Intervention

**DOI:** 10.3389/fonc.2020.01242

**Published:** 2020-07-22

**Authors:** Stefano Falone, Athanassios Vassilopoulos, Lucia Altucci

**Affiliations:** ^1^Department of Life, Health and Environmental Sciences, University of L'Aquila, L'Aquila, Italy; ^2^Department of Radiation Oncology, Northwestern University, Chicago, IL, United States; ^3^Robert H. Lurie Comprehensive Cancer Center, Feinberg School of Medicine, Northwestern University, Chicago, IL, United States; ^4^Department of Precision Medicine, University of Campania “Luigi Vanvitelli”, Naples, Italy

**Keywords:** sirtuins, cancer, chemoresistance, malignant phenotype, chemotherapy, tumor, SIRT

Extensive reprogramming of energy metabolism and detoxification processes are increasingly seen as critical factors involved in metastatic progression and in development of chemo- and radio-resistance (1–3). Mammal sirtuins (SIRT1-7) are a family of conserved NAD^+^-dependent protein deac(et)ylases and/or mono-[ADP-ribosyl]transferases with varied cellular distribution. Their role as epigenetic players and crucial regulators of energy metabolism and adaptation to cellular stress is currently under extensive investigation worldwide, not only in physiological processes (e.g., in aging) but also in the pathogenesis of cardiovascular and neurodegenerative diseases, diabetes and cancer (1, 2, 4–6). In particular, sirtuin-dependent signaling is suspected to play a dual role in cell biology, on one hand protecting DNA from genomic instability and limiting the replicative potential, on the other hand inhibiting senescence and promoting survival and growth advantage (7). Interestingly, SIRT3-5 localize to mitochondria and regulate targets involved in diverse biomolecular pathways, including energy metabolism and apoptotic death (8–11). Such characteristics confer a great importance to sirtuins, in terms of preventive medicine and therapeutic potential in anticancer strategies.

Unfortunately, despite the broad interest in this field, results currently available are still insufficient to draw definitive conclusions about the role of the sirtuinome in the regulation of key aspects of tumor cell biology, as well as of the interactions between cancer cells and the surrounding environment. More importantly, the key question as to whether sirtuins can be considered as tumor suppressors or oncogenic proteins remains unanswered.

In this Research Topic we collected original studies (mini)review and perspective articles that were focused on the SIRT-dependent mechanisms that underlie various tumor- and cancer-related processes, both at cellular and tissue level.

Solid tumors are often accompanied by neo-vascularization that is needed to create a highly integrated micro-ecosystem aimed at limiting both hypoxic stress and build-up of toxic tumor metabolites. As reviewed by Edatt et al., sirtuins seem to play an important role in the regulation of the functional cross-talk between pro-angiogenic and anti-angiogenic signaling surrounding neoplasms. This is achieved by controlling proliferation and migration of endothelial cells, as well as through the direct or indirect modulation of the activity of eNOS, p53, HIF-1α, FOXO, Notch, VEGF, and other factors that are essential to vascular function and organization. The authors also provided evidence of the involvement of several key miRNAs in such a regulation. Edatt et al. gave also interesting details of the crucial cross-talk between pro-inflammatory signaling and pro-angiogenic pathways controlled by sirtuins in the tumor milieu, linking SIRT-dependent changes in NFκB signal transduction to interleukin release. The authors concluded that despite some apparent contradicting angiogenic roles seemingly played by sirtuins in tumors, the SIRT-dependent epigenetic regulation of vascular remodeling is increasingly considered as a promising therapeutic target to limit or prevent tumor angiogenesis.

Schwartsburd hypothesized a mechanism by which upon metabolic/hormonal changes may provoke glucose delivery to cancer cells via two interconnected “vicious cycles” driving cancer progression. The proposed “vicious cycles” result from cancer-mediated manipulation of host glucose sensors. The author concludes that this knowledge may help in identifying novel potential therapeutic targets. Tomaselli et al. provide an overview of the role of the mitochondrial sirtuin 4, whose deregulation is connected to aging-related disorders. In addition to its initial described role as a mono ADP-ribosyltransferase, several other enzymatic and non-enzymatic activities have been described which support its critical role in regulating mitochondrial metabolism. Despite the progress in this area, how SIRT4 affects cancer remains controversial. Both pro and anti-tumorigenic activities have been reported which emphasize the significant contribution of factors such as tumor type, stage, and biological context that need to be further elucidated. Ahmed et al. have focused their efforts on uncovering the roles of SIRT2 and SIRT3 *in vivo* in mice under caloric restriction (CR) or fed a high-fat diet (HFD). Interestingly, they report that the anti-cancer effect of CR was not observed in the Sirt2^−/−^ mice, while Sirt3^−/−^ mice exhibited protection against the tumor promoting effect of HFD. Considering that only SIRT1 has been studied so far in this context, this study provides novel insights regarding the role of two other sirtuin family members in tumorigenesis under CR or HFD. Carafa et al. hypothesized that targeting mitochondrial function might be a possible anticancer strategy. Indeed, the authors pointed out that the treatment with a novel pan Sirt inhibitor might orchestrate cell response to metabolic stress interfering with cancer progression. Finally, Gaál and Csernoch critically review the impact of sirtuin enzymes on the altered metabolic phenotype of malignantly transformed cells. The authors describe in depth the impact of sirtuins on the epigenetic and metabolic alterations in malignancy. Despite the best-known metabolic features are augmented glycolysis and lactate production, Warburg effect also includes carbohydrate, lipid, and amino acid metabolism. The authors also discuss how the SIRT-dependent alteration of metabolism in cancer cells has an impact on changes in gene expression pattern within the tissue microenvironment.

The topic editorial board sincerely hopes that the articles collected in this Research Topic may give a significant contribution to the knowledge of how SIRTs are able to drive molecular adaptations and phenotype changes in tumors and malignancies, thus unveiling important pathways and potential therapeutic targets of clinical relevance.

## Author Contributions

SF, AV, and LA wrote, reviewed, and approved the content of this editorial. All authors contributed to the article and approved the submitted version.

## Conflict of Interest

The authors declare that the research was conducted in the absence of any commercial or financial relationships that could be construed as a potential conflict of interest. The handling editor declared a past co-authorship with one of the author SF.
